# Acute partial Budd-Chiari syndrome and portal vein thrombosis in cytomegalovirus primary infection: a case report

**DOI:** 10.1186/1471-230X-6-10

**Published:** 2006-03-10

**Authors:** Laurent Spahr, Andreas Cerny, Isabelle Morard, Laura Rubbia-Brandt, Jacques Schrenzel

**Affiliations:** 1 Gastroenterology and Hepatology, Ospedale Regionale, Lugano, Switzerland; 2 Servicio di Medicina Interna, Ospedale Regionale, Lugano, Switzerland; 3 Clinical Pathology, University Hospital, Geneva, Switzerland; 4 Infectious Diseases, University Hospital, Geneva, Switzerland

## Abstract

**Background:**

Splanchnic vein thrombosis may complicate inherited thrombotic disorders. Acute cytomegalovirus infection is a rare cause of acquired venous thrombosis in the portal or mesenteric territory, but has never been described extending into a main hepatic vein.

**Case presentation:**

A 36-year-old immunocompetent woman presented with acute primary cytomegalovirus infection in association with extensive thrombosis in the portal and splenic vein. In addition, a fresh thrombus was evident in the right hepatic vein. A thorough evaluation for a hypercoagulable state was negative. The clinical course, biological evolution, radiological and histological findings were consistent with cytomegalovirus hepatitis complicated by a partial acute Budd-Chiari syndrome and portal thrombosis. Therapeutic anticoagulation was associated with a slow clinical improvement and partial vascular recanalization.

**Conclusion:**

We described in details a new association between cytomegalovirus infection and acute venous thrombosis both in the portal vein and in the right hepatic vein, realizing a partial Budd-Chiari syndrome. One should be aware that this rare thrombotic event may be complicated by partial venous outflow block.

## Background

The development of venous thrombosis has been occasionally reported in the setting of acute cytomegalovirus infection in immunocompetent adults, possibly as a result of a procoagulant effect on endothelial cells. Such a vascular event may take place in the splanchnic territory (portal and mesenteric vein), but has never been described in the hepatic veins.

Hepatic vein obstruction is associated to a clinical and pathological presentation named the Budd-Chiari syndrome, including hepatic sinusoidal dilatation and congestion, ascites and abdominal pain, as a result of hepatic outflow block. Hepatic vein thrombosis, typically associated to primary myeloproliferative disorders, has never been described in association with an acute infection. We provide here a detailed observation of a partial acute Budd-Chiari syndrome complicating an acute cytomegalovirus infection.

## Case presentation

A 36-year-old woman presented with abdominal pain and fever (38.5–39°C) of 3 weeks duration. She was previously healthy, and was on oral oestroprogestative since one year. Two years ago, CMV IgG tested negative. Leukocyte count was 9700 cells/mm^3^, platelets were 80000/mm^3^, and C-reactive protein level was 110 mg/L (N: < 10). ALT level was 49 U/L (N: < 37), AST was 45 U/L (N: < 36), γ-glutamyltranspeptidase level was 49 (N: < 36), with normal LDH, alkaline phosphatase, bilirubin and INR values. Haptoglobin value was 2.06 g/l (N: 0.28–1.78). Prothrombin time and fibrinogen level were normal. An abdominal CT scan showed an enlarged spleen and a thrombus in the splenic vein extending into the portal trunk. A fresh thrombus was also evident in the right hepatic vein (see figure). A thorough evaluation for a hypercoagulable state was performed: homocystein level: 6.7 μmol/l (N: < 15); antithrombin III: 86% (N: 75–125); protein C: 74% (N: 70–140); protein S: 88% (N: 50–120); factor V Leiden and prothrombin 20210 genes: not mutated; anticardiolipin antibodies: negative; antinuclear antibodies: < 1/60; paroxysmal nocturnal hemoglobinuria: absence of deficience of GPI-linked proteins by flow cytometry. Although a bone marrow analysis was not performed, an overt myeloproliferative disorder was deemed unlikely in the absence of grossly elevated white blood cells and platelet count, no eosinophilia, basophilia, or atypical platelet morphology on the peripheral smear examination. We felt that the probability of an early hepatocellular carcinoma in the absence of a pre-existing liver disease was extremely low. The value of AFP was 4 ng/ml (N < 15 ng/ml)

Blood cultures remained sterile, and serological tests for hepatitis A, B, C and HIV were negative. Serum immunoglobulins were as follows: IgG: 19 g/l (N: 6.7–13); IgA: 4.3 g/l (N: 0.7–2.9); IgM: 2.6 (N: 0.4–2.6). Typisation of circulating lymphocytes (T, B, NK) showed normal values.

Serological testing for CMV showed elevated titers of IgG and IgM. CMV DNA using real-time PCR was detectable at a level of 5 copies per 10^6^, with a partial inhibition of amplification. EBV DNA was negative. Mild duodenal inflammation was observed at endoscopy, with viral-type inclusions visible in endothelial cells seen in intestinal biopsy.

Intravenous heparin at a therapeutic level was administered, and oral contraception was stopped. The patient remained febrile (temperature 39°C), and the abdominal pain increased in intensity despite full-dose anticoagulation. The AST and ALT levels increased (76 U/L and 111 U/L, respectively). A repeat abdominal CT scan showed moderate ascites. Using a transjugular approach, direct venography was performed after a difficult cannulation of the right hepatic vein, demonstrating a "spider-web" network pattern typically seen in Budd-Chiari syndrome (see figure). The hepatic venous pressure gradient showed moderate portal hypertension, with a value of 9 mmHg (N: < 4). Liver histology (see figure) showed acute hepatitis with a mononuclear and neutrophilic infiltrate, scattered areas of hepatocyte necrosis, and a microgranulomatous cell reaction. An immunohistochemistry study was performed on the liver biopsy specimen to detect CMV[1], but remained negative. There were no features of a pre-existing liver disease, and no lesions (plasma cells infiltration, interface hepatitis) that may suggest the presence of auto-immune hepatitis[2]. In addition, a marked sinusoidal dilatation and congestion in the centrilobular regions were consistent with a hepatic vein outflow obstruction[3].

Five days after liver biopsy, transaminases returned to normal values. The patient remained febrile with mild abdominal pain persisting another 3 weeks. At 5 months, control ultrasonography showed persistently obstructed right hepatic, splenic and portal vein with a cavernoma, but no ascites.

## Conclusion

Splanchnic thrombosis in association with acute CMV infection is a very rare event in an immunocompetent host[4]. It has been reported in the portal or mesenteric territory [5-7] and in extra abdominal vascular beds[8], sometimes associated with an underlying prothrombotic state. We describe here a detailed case of acute CMV infection with typical microgranulomatous hepatitis and splanchnic thrombosis, and report for the first time the development of an acute thrombosis in a main hepatic vein consistent with a partial Budd-Chiari syndrome.

Cytomegalovirus infection in an immunocompetent host may occur in childhood or in young adulthood, and often follows an asymptomatic course. In an immunocompromized patient, this important viral pathogen may induce serious complications including colitis, esophagitis or pneumonitis[9]. In this young immunocompetent female adult, the diagnosis of acute CMV primary infection was established on a positive viremia and a typical acute microgranulomatous hepatitis[10]. Clinical features included a severe mononucleosis-like syndrome and an acute thrombosis in the splenic, portal and right hepatic vein. Although fatal issues have been reported in severe cytomegalovirus infection in such individuals[4], we decided not to administer ganciclovir to our patient in the view of a relatively modest viral load, preserved white blood cell count and absence of detectable CMV on livr biopsy. Accordingly, antiviral treatment is not routinely recommended in immunocompetent individuals with severe CMV infection[4].

The radiological evidence of hepatic vein thrombosis, an enlarged liver, an increased abdominal pain, congestion around the central vein of the liver lobule at histology, elevated portal pressure, raised serum aminotransferases and presence of ascites rendered the diagnosis of partial Budd-Chiari syndrome most likely[11]. Due to the limited extent of the hepatic venous thrombosis, clinical and biological alterations were limited in intensity[3]. Thus, thrombolysis or mechanical angioplasty were not considered in the present case.

Although splanchnic vein thrombosis may result from a combination of several factors, it is currently indicated to search for an underlying hypercoagulable disorder[11]. In our patient, we did not identify a coagulation disorder, but we hypothesized that the acute CMV infection combined with oral contraceptives precipitated the acute and extensive vascular thrombosis. *In vitro*, CMV infection induces a procoagulant state by altering endothelial cell surface[12]. The alteration in the endothelium phenotype that includes overexpressed surface adhesion molecules[13] stimulates leucocytes and platelets adhesion[14] and results in an increased procoagulant activity[15]. Thus, a number of observations point towards a damage to vascular endothelium in the presence of CMV, and supports the hypothesis that acute CMV infection increases the risk of thrombosis.

In conclusion, CMV primary infection, i) should be considered in the diagnostic work-up of acute splanchnic vein thrombosis, and ii) may be associated to a partial Budd-Chiari syndrome.

## Abbreviations

CMV: cytomegalovirus infection

## Competing interests

The author(s) declare that they have no competing interests.

## Authors' contributions

L.S. performed the invasive procedure, and wrote and discussed the case report

I.M: coordinated the coagulation work-up and clinical follow-up

L.R-B: performed the histopathological analysis

J.S: coordinated the virological studies

## Pre-publication history

The pre-publication history for this paper can be accessed here:



## Figures and Tables

**Figure 1:**
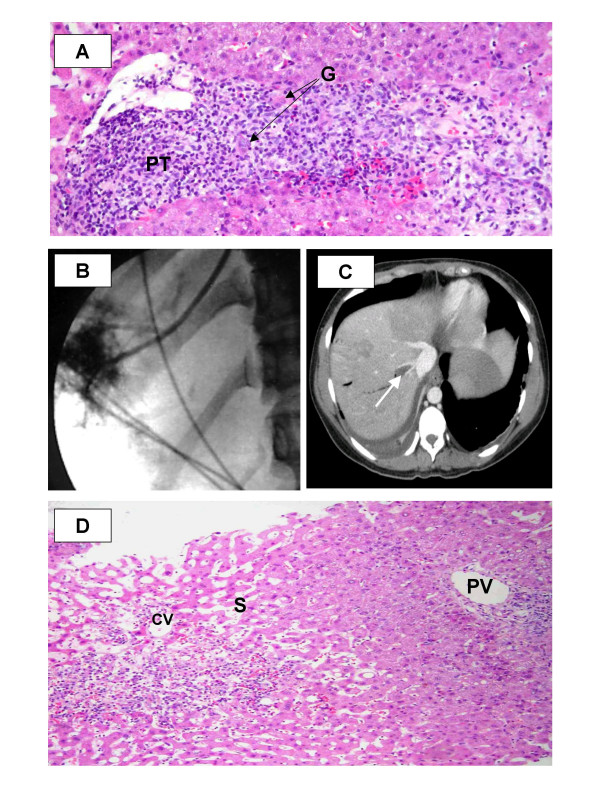
Panel A: Low magnification of the liver biopsy (HE 120 ×). The portal tract (PT) is infiltrated by a mixed inflammatory reaction. Microgranulomatous cell reaction (G) is visible. Panel B: Typical "spider-web" network pattern at hepatic angiography in the setting of partial Budd-Chiari syndrome. Panel C: Abdominal CT scan showing a fresh thrombus in the ostium of the right hepatic vein (arrow). Panel D: Low magnification of liver biopsy showing marked sinusoidal dilatation (S) and congestion around the central vein (CV). PV: portal vein.
